# Intermuscular Adipose Tissue as a Multimodality Quantitative Imaging Biomarker: Evidence Across Metabolic, Cardiovascular and Oncologic Diseases

**DOI:** 10.1002/jcsm.70366

**Published:** 2026-08-17

**Authors:** Feier Ding, Atsushi Nakamoto, Xiaohan Zheng, Toru Honda, Gongzheng Wang, Yuwei Liu, Hideyuki Fukui, Takashi Ota, Masahiro Yanagawa, Noriyuki Tomiyama

**Affiliations:** ^1^ Department of Radiology The University of Osaka Graduate School of Medicine Suita Osaka Japan; ^2^ Department of Radiology The Fourth Central Clinical School, Tianjin Medical University Tianjin China; ^3^ Department of Radiology Shandong Provincial Hospital Affiliated to Shandong First Medical University Jinan Shandong China; ^4^ Laboratory for Theranostics, Division of Clinical Translation, Institute for Radiation Sciences The University of Osaka Graduate School of Medicine Suita Osaka Japan

## Abstract

Intermuscular adipose tissue (IMAT) refers to adipose tissue located between muscle groups or beneath the muscle fascia, and its accumulation is closely linked to impaired muscle quality, reduced contractile function, and adverse metabolic profiles, establishing it as a core component of myosteatosis. Unlike subcutaneous and visceral adipose tissue, which reside outside the muscle architecture, IMAT expands within the muscle environment, where local metabolic and mechanical interactions may affect muscle integrity and performance. Elevated IMAT has been associated with diminished strength, impaired mobility, frailty, insulin resistance, and adverse cardiometabolic profiles, underscoring its clinical relevance beyond a passive fat depot. However, IMAT evidence remains dispersed across imaging techniques, anatomical regions, and disease‐specific literatures, limiting a unified understanding of its clinical utility. This review synthesizes current evidence on IMAT biology, imaging‐based quantification, disease‐specific distribution and clinical implications, with emphasis on its value as a risk‐stratification biomarker. Recent advances in medical imaging, including computed tomography (CT), magnetic resonance imaging (MRI), ultrasound and positron emission tomography (PET), have enabled noninvasive and reproducible IMAT quantification. These modalities provide complementary measures of muscle fat infiltration, including CT‐derived area, volume and attenuation; MRI‐based segmentation and proton density fat fraction; ultrasound echo intensity; and PET‐derived metabolic activity. Deep learning–based segmentation methods have improved large‐scale IMAT quantification and reproducibility across anatomical regions. Mechanistically, IMAT is associated with chronic low‐grade inflammation and impaired glucose homeostasis, promoting progressive fat infiltration and muscle degeneration. Human studies indicate that IMAT exhibits an inflammatory secretory profile linked to insulin resistance and metabolic dysfunction. Quantitative imaging shows anatomically heterogeneous IMAT distribution, with frequent involvement of the lower extremities, paraspinal muscles and trunk. Across disease settings, IMAT appears to provide complementary information beyond muscle mass alone by capturing muscle quality and systemic vulnerability. Evidence is strongest in oncology, where elevated IMAT or IMAT‐based ratios have repeatedly been associated with poorer survival, recurrence, treatment toxicity, and reduced treatment tolerance. In metabolic and musculoskeletal disorders, IMAT is consistently related to insulin resistance, reduced strength, impaired mobility, and frailty, whereas cardiovascular evidence suggests prognostic relevance but remains less standardized and more heterogeneous. Clinically, IMAT imaging may help identify patients whose preserved muscle size or body mass index masks poor muscle quality, thereby refining prognostic assessment, perioperative risk evaluation, and selection for nutritional, exercise, metabolic, or treatment strategies. Future efforts should focus on standardized acquisition, automated segmentation, harmonized thresholds, and prospective validation to support integration of IMAT metrics into clinical decision‐making.

**Abbreviations**
AI: artificial intelligence
BMI: body mass index
COPD: chronic obstructive pulmonary disease
CRP: C‐reactive protein
CSA: cross‐sectional area
CSE‐MRI: chemical shift‐encoded magnetic resonance imaging
CT: computed tomography
DWI: diffusion‐weighted imaging
FAP: fibro‐adipogenic progenitor
FDG: fluorodeoxyglucose
GOLD: Global Initiative for Chronic Obstructive Lung Disease
HCC: hepatocellular carcinoma
HU: Hounsfield unit
ICU: intensive care unit
IDEAL: iterative decomposition of water and fat with echo asymmetry and least‐squares estimation
IL‐6: interleukin‐6
IMAT: intermuscular adipose tissue
kVp: kilovoltage peak
L3: third lumbar vertebral level
LAMA: low‐attenuation muscle area
MCP‐1: monocyte chemoattractant protein‐1
MRI: magnetic resonance imaging
mRNA: messenger RNA
MrOS: Osteoporotic Fractures in Men Study
MT: muscle thickness
MVI: microvascular invasion
PDFF: proton density fat fraction
PET: positron emission tomography
PET/CT: positron emission tomography/computed tomography
PET/MRI: positron emission tomography/magnetic resonance imaging
pQCT: peripheral quantitative computed tomography
PSMA: prostate‐specific membrane antigen
ROI: region of interest
SAT: subcutaneous adipose tissue
SGLT2: sodium–glucose cotransporter 2
SMD: skeletal muscle density
SUVmax: maximum standardized uptake value
SUVmean: mean standardized uptake value
T12: twelfth thoracic vertebral level
T2DM: type 2 diabetes mellitus
TAT: total adipose tissue
TGF‐β1: transforming growth factor‐beta 1
TNF‐α: tumour necrosis factor‐alpha
VAT: visceral adipose tissue

## 1 Introduction

**FIGURE 1:**
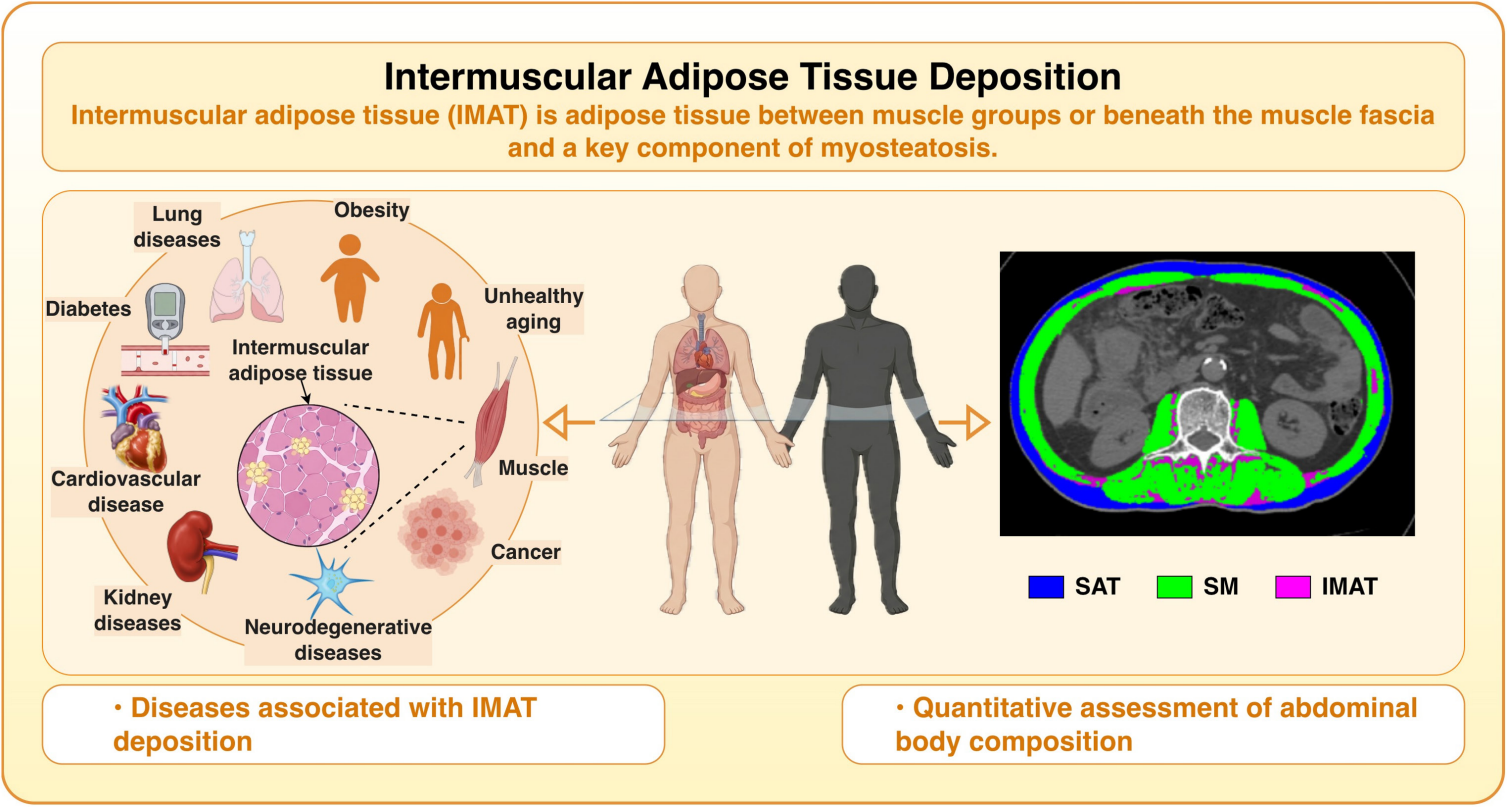
Overview of IMAT deposition and its associated clinical implications, with a representative CT image at the L3 level showing segmentation of SAT, SM and IMAT as an example of quantitative body composition analysis; IMAT can also be assessed in other muscle groups depending on the study context. CT = computed tomography; IMAT = intermuscular adipose tissue; L3 = third lumbar vertebral level; SAT = subcutaneous adipose tissue; SM = skeletal muscle. (Created with BioRender.com).

Intermuscular adipose tissue (IMAT) refers to adipose tissue located between muscle groups or beneath the muscle fascia, and its accumulation is closely linked to impaired muscle quality, reduced contractile function, and adverse metabolic profiles, establishing it as a core component of myosteatosis [1, 2, 3, 4]. In contrast to intramyocellular lipids within myocytes and extramyocellular lipids positioned outside muscle fibres but within the fascial compartment, IMAT occupies interstitial spaces between muscle bundles and integrates into the structural framework of skeletal muscle [5]. Subcutaneous adipose tissue (SAT) and visceral adipose tissue (VAT) reside outside the muscle architecture, whereas IMAT expands within the muscle environment where local metabolic and mechanical interactions exert direct effects on muscle integrity and performance [6, 7, 8] (Figure 1). More broadly, skeletal muscle is increasingly recognized as an organ involved in the pathophysiology of numerous acute and chronic diseases. Accordingly, its clinical assessment should extend beyond muscle quantity to muscle composition, as excessive lipid accumulation within skeletal muscle, including expansion of IMAT, represents myosteatosis and may reflect pathological muscle remodelling [9].

Elevated IMAT has been consistently linked to diminished muscle strength, impaired balance, and functional decline in aging populations [10, 11, 12]. Increasing IMAT accompanies reductions in muscle mass and mobility and contributes to frailty and disability [13, 14]. Specifically, pro‐inflammatory cytokines such as tumour necrosis factor‐alpha (TNF‐α) and interleukin‐6 (IL‐6) are elevated in IMAT‐rich muscle, driving insulin resistance and altering fibro‐adipogenic progenitor (FAP) behaviour, which promotes further adipogenic differentiation and fat infiltration [13, 15, 16]. Mitochondrial dysfunction, reduced oxidative capacity, and impaired satellite‐cell–mediated regeneration further compromise muscle repair capacity and promote progressive myosteatosis [13]. IMAT is not necessarily completely absent at birth, as fibro‐adipogenic lineages are established during prenatal skeletal muscle development [17]. However, direct quantitative evidence in healthy human neonates remains limited. IMAT undergoes postnatal remodelling and generally accumulates with aging, physical inactivity, metabolic dysfunction, chronic inflammation, and impaired muscle regeneration [13, 17, 18]. Under these conditions, dysregulated FAPs promote adipogenesis, fibrosis and progressive fatty infiltration of skeletal muscle [13, 18].

In recent years, medical imaging advancements (computed tomography [CT], magnetic resonance imaging [MRI], ultrasound and positron emission tomography [PET]) have enabled noninvasive quantification of IMAT in both clinical studies and animal models. CT distinguishes IMAT from muscle by tissue density differences (Hounsfield units, HUs), whereas MRI provides a gold‐standard quantification using techniques such as Dixon or proton density fat fraction (PDFF) [19, 20, 21]. CT and MRI are generally regarded as in vivo reference methods for assessing skeletal muscle mass and composition, although standardized acquisition protocols, analytical methods, and terminology remain necessary [9]. Ultrasound facilitates bedside evaluation of muscle composition, and PET can characterize metabolic activity in fatty‐infiltrated adipose tissue. Deep‐learning segmentation approaches have improved large‐scale IMAT quantification and strengthened reproducibility across heterogeneous anatomical regions [22, 23]. Importantly, imaging studies have demonstrated that IMAT accumulation is strongly associated with insulin resistance, type 2 diabetes mellitus (T2DM), chronic back pain and sarcopenia, highlighting its clinical value as a biomarker of metabolic dysfunction and musculoskeletal decline [3, 24].

Against this background, the present review synthesizes three central themes: how IMAT is best assessed using modern imaging techniques; how IMAT distribution and imaging patterns differ across metabolic disease, age‐associated decline, and cancer‐related states; and how IMAT‐derived imaging metrics can inform clinical decision‐making. Previous high‐quality reviews have addressed IMAT mainly in the contexts of metabolic disease, myosteatosis, sarcopenia, or general skeletal muscle and body‐composition methodology [1, 9, 11, 25]. However, evidence remains fragmented across imaging modalities, anatomical regions, disease‐specific bodies of literature, and clinical endpoints. The distinctive contribution of this review is therefore an imaging‐centered, cross‐disease synthesis of IMAT as a quantitative biomarker. We integrate mechanistic evidence, modality‐specific imaging approaches, anatomical distribution, and disease‐specific prognostic findings to clarify its potential role in clinical risk stratification and treatment monitoring.

## 2 Mechanistic Foundations Linking IMAT and Disease Pathophysiology

IMAT is associated with impaired glucose homeostasis, including higher fasting glucose, reduced glucose tolerance, and lower insulin sensitivity. Human experiments show that IMAT conditioned media reduces insulin sensitivity in primary myotubes with potency similar to visceral fat, whereas subcutaneous fat media shows little effect. Compositional profiling reveals that human IMAT secretes a distinct, inflammatory secretome of cytokines, chemokines, adipokines and eicosanoids linked to insulin resistance [26]. IMAT abundantly expresses inflammatory mediators such as monocyte chemoattractant protein‐1 (MCP‐1), IL‐6 and TNF‐α, and evidence indicates upregulation of these signals at both messenger RNA (mRNA) and protein levels. In addition, transforming growth factor‐beta 1 (TGF‐β1) is strongly expressed in IMAT‐resident FAPs and regulates their cellular differentiation [13, 18, 26]. At the cellular level, FAPs, which are the primary progenitors of IMAT adipocytes, are highly sensitive to inflammatory stimuli; for example, TNF‐α promotes FAP apoptosis through death receptor pathways. Together, these findings indicate that IMAT is not simply a secondary marker of metabolic dysfunction but may actively contribute to chronic inflammation and metabolic disease. Accumulating evidence also indicates that IMAT is a dynamic tissue that responds to systemic inflammation, physical inactivity, and metabolic stress, which may initiate or accelerate muscle atrophy [13, 18]. IMAT accumulation correlates independently with reduced muscle strength, impaired mobility, and adverse cardiometabolic profiles, underscoring its importance as an active mediator of musculoskeletal decline.

## 3 Imaging‐Based Assessment and Quantification of IMAT

### 3.1 Imaging Modalities Development

Before comparing individual imaging modalities, it is important to distinguish IMAT metrics according to the tissue property being measured. Macroscopic IMAT area or volume represents spatially resolvable adipose tissue located within the deep fascial boundary, predominantly between adjacent muscle groups [27, 28]. In contrast, CT‐derived muscle attenuation and MRI‐derived PDFF characterize overall muscle composition or diffuse fatty infiltration and should not be interpreted as direct equivalents of anatomically segmented IMAT [21, 28, 29, 30, 31]. Ultrasound echo intensity provides an indirect marker of altered muscle composition [25, 32], whereas PET primarily evaluates metabolic activity rather than IMAT quantity [33, 34, 35]. Accordingly, CT, MRI, ultrasound and PET provide complementary rather than interchangeable measures of IMAT‐related muscle characteristics.

Among these modalities, CT has been most widely used for the anatomical assessment of IMAT because it enables adipose tissue to be distinguished from lean muscle based on differences in X‐ray attenuation. Current imaging approaches for IMAT assessment are summarized in Table 1, including the main modalities, technical methods, and clinical applications. CT imaging enables precise identification of IMAT by leveraging the attenuation differences between fat and lean tissue. Adipose tissue typically has CT attenuation values between −190 and −30 HU [27], allowing it to be distinguished from other body composition compartments. Early population studies showed that thigh IMAT relates to insulin resistance in older adults independent of overall and visceral adiposity [4] and that low subcutaneous thigh fat predicts adverse glucose and lipid profiles. However, CT‐derived muscle metrics are sensitive to technical parameters such as tube voltage (kVp), contrast agent usage, and slice thickness. Recent studies using opportunistic CT at the L3 vertebral level have demonstrated IMAT is strongly associated with skeletal muscle density (SMD) [29]. The L3 level has become a pragmatic standard in CT‐based body composition analysis not because IMAT is confined to the psoas or paraspinal muscles, but because a single axial image at this level provides a reproducible and widely validated estimate of abdominal skeletal muscle and adipose tissue compartments. In oncologic imaging, L3 is also frequently available on routine abdominal CT, allowing opportunistic quantification without additional imaging acquisition [10, 27]. Nevertheless, L3‐based assessment should be interpreted as a standardized surrogate rather than a complete representation of whole‐body IMAT burden. This is particularly important because IMAT distribution is anatomically heterogeneous, with substantial deposition reported in lower‐extremity muscle groups such as the thigh and calf [4, 44]. Among the variables examined, IMAT shows the strongest standardized effect on SMD, highlighting its dominant role in reducing muscle density. Peripheral quantitative CT (pQCT) is another CT‐based modality relevant to IMAT assessment, particularly in limb‐focused musculoskeletal cohorts. Standardized pQCT scans of the lower leg, tibia, forearm, or mid‐arm can quantify muscle cross‐sectional area (CSA) and muscle density, with reduced muscle density serving as an indirect marker of intra‐ and intermuscular fat infiltration. However, pQCT has limited anatomical coverage and cannot reliably delineate fascial boundaries, so marked fatty infiltration may cause segmentation errors or require manual/watershed‐based correction [41]. Despite its strengths, such as high spatial resolution and the potential for large‐scale, opportunistic analyses, CT imaging also presents limitations. It involves ionizing radiation, which restricts its use in paediatric populations and repeated assessments. Moreover, CT lacks chemical specificity, making it difficult to distinguish between intermuscular and intramyocellular fat solely based on attenuation values [30]. Overall, CT‐based modalities have greatly enhanced our ability to assess IMAT, but further development is needed to improve standardization, reduce radiation exposure, and incorporate techniques that can chemically differentiate fat compartments within muscle tissue.

**TABLE 1:** Imaging‐based assessment of IMAT: modalities, techniques and key findings.

Modality	Participants/setting	IMAT assessment method	Outcome measures/applications	Key references
CT	MrOS musculoskeletal cohort	U‐Net segmentation	Strength; mobility	[23]
	Early IMAT studies	Fat attenuation −190 to −30 HU	Automated IMAT detection	[27]
	Older adults' metabolic cohort	Thigh IMAT quantification	Insulin resistance; glucose/lipid profiles	[4]
	Opportunistic L3 abdominal CT	L3 IMAT measurement	SMD association	[29]
	Metastatic prostate cancer	IMAT/TAT, IMAT/SAT ratios	Survival prediction	[36]
	PAD patients (CTA)	IMAT fraction on CT angiography	Amputation risk prediction	[37]
	Chronic liver	Radiomics integrating IMAT/VAT/SAT	Hepatic decompensation	[38]
	Cardiometabolic	IMAT density metrics	Major adverse cardiovascular outcomes	[39, 40]
	Peripheral musculoskeletal cohorts using pQCT	Muscle CSA and density	Limb muscle composition; indirect fat‐infiltration marker; limited coverage	[41]
MRI	Whole‐body MRI	Deep learning–based IMAT quantification	Association with glucose metabolism	[42]
	Body composition MRI	Dual‐parametric MRI + hierarchical U‐Net (BioCompNet)	Automated IMAT quantification	[43]
	Chronic pain cohorts	Whole‐body IMAT MRI	Chronic musculoskeletal pain	[24]
	Longitudinal imaging cohorts	Repeated IMAT quantification	Disease/weight‐change remodelling	[44]
	Histology validation cohort	MRI IMAT segmentation	MRI–histology concordance	[45]
Ultrasound	Critical care; geriatrics; rehabilitation	EI	Muscle quality; fibrosis	[25, 32]
	Sarcopenia/cachexia; rehabilitation; bedside assessment	Shear‐wave elastography	Emerging marker of muscle stiffness and muscle quality	[46]
	Sports/rehabilitation/clinical	CSA and MT	Structural IMAT evaluation	[47, 48]
	ICU monitoring	IMAT‐index (EI + CSA)	Real‐time IMAT estimation	[49]
PET/CT or PET/MRI	Hybrid imaging; IMAT metabolic studies	PET/CT or PET/MRI fusion imaging	Structural + metabolic characterization	[33]
	Oncologic cohorts	^18^F‐FDG uptake and CT‐based IMAT segmentation	Metabolic characterization; prediction of lymphovascular invasion and survival	[34, 35, 50]

Abbreviations: ^18^F‐FDG = fluorine‐18 fluorodeoxyglucose; BioCompNet = deep learning–based body‐composition analysis framework; CSA = cross‐sectional area; CT = computed tomography; CTA = computed tomography angiography; EI = echo intensity; HU = Hounsfield unit; ICU = intensive care unit; IMAT = intermuscular adipose tissue; L3 = third lumbar vertebral level; MRI = magnetic resonance imaging; MrOS = Osteoporotic Fractures in Men Study; MT = muscle thickness; PAD = peripheral arterial disease; PET/CT = positron emission tomography/computed tomography; PET/MRI = positron emission tomography/magnetic resonance imaging; pQCT = peripheral quantitative computed tomography; SAT = subcutaneous adipose tissue; SMD = skeletal muscle density; TAT = total adipose tissue; U‐Net = U‐shaped convolutional neural network; VAT = visceral adipose tissue.

MRI has become an indispensable tool for visualizing IMAT, offering non‐invasive, radiation‐free imaging with superior soft tissue contrast. Conventional T1‐weighted MRI enables segmentation‐based quantification of IMAT area and volume through signal‐intensity thresholding techniques [28]. In contrast, chemical shift‐encoded water‐fat MRI (CSE‐MRI), derived from the Dixon method, allows quantitative assessment of muscle fat infiltration by measuring PDFF. Advanced CSE‐MRI methods, such as multipeak iterative decomposition of water and fat with echo asymmetry and least‐squares estimation (IDEAL), improve water‐fat separation by accounting for the multipeak spectral characteristics of fat [31]. Whole‐body MRI quantification of IMAT has shed light on its potential systemic impact. Recent deep learning‐based whole‐body MRI studies have demonstrated that automated body composition analysis can be performed at scale and can provide volumetric or regional measurements of muscle and adipose compartments, including IMAT‐related phenotypes [22, 42, 43]. However, MRI‐based IMAT quantification is limited by longer acquisition time, higher cost, motion artefacts and protocol heterogeneity across scanners, sequences and post‐processing pipelines, which may reduce comparability across centers. These advances indicate a methodological shift from single‐slice cross‐sectional analysis toward multi‐regional and whole‐body assessment. However, whole‐body volumetric IMAT quantification has not yet become a universally standardized clinical approach, partly because acquisition protocols, anatomical boundaries, segmentation labels, and normalization strategies differ across studies. Therefore, future research should prioritize harmonized definitions and reproducible volumetric pipelines that can distinguish anatomically segmented IMAT from diffuse muscle fat fraction measured by PDFF.

Beyond structural imaging, multiparametric MRI—including diffusion‐weighted imaging (DWI) and CSE‐MRI—allows for enhanced characterization of IMAT by providing insights into its metabolic activity and fat content. The clinical credibility of MRI‐based IMAT quantification is well supported by its strong concordance with histological analyses [45]. However, achieving consistent real‐world deployment requires overcoming several practical constraints, such as standardization of acquisition protocols, high costs and motion artefacts [28]. Promising solutions include artificial intelligence (AI)‐based segmentation and accelerated MRI protocols.

Ultrasound imaging enables IMAT assessment through several key techniques. Echo intensity analysis quantifies grayscale histogram data or mean pixel brightness as an indirect marker of fat infiltration and fibrosis [25, 32]. Shear‐wave elastography is an emerging ultrasound‐based method for assessing muscle quality through muscle stiffness or shear‐wave speed, but its relationship with myosteatosis remains inconsistent and requires further validation across muscles and clinical populations [46]. CSA and muscle thickness (MT) are measured in transverse or longitudinal planes to evaluate muscle quantity and estimate relative fat proportions [47, 48]. Automated IMAT indices, such as the MuscleSound IMAT‐Index, integrate echo intensity and CSA to provide real‐time estimates of the relative intramuscular adipose tissue content, supporting continuous monitoring in dynamic clinical settings like the intensive care unit (ICU) [49]. Nevertheless, ultrasound‐derived echo intensity is operator‐ and device‐dependent and can be influenced by probe pressure, gain settings, subcutaneous fat thickness, and limited visualization of deep muscle groups. These challenges are amplified when assessing deeper or larger muscles, such as the psoas and paraspinal muscles. Variability in acquisition protocols, device settings, region of interest (ROI) placement and echo‐intensity thresholds further limits cross‐study comparability and clinical standardization.

PET/CT or PET/MRI fusion imaging combines the anatomical resolution of CT or MRI with the metabolic assessment of PET, offering a powerful tool to investigate the metabolic characteristics of IMAT [33]. In a recent PET/CT–based body composition analysis, Jiang et al. reported that IMAT maximum standardized uptake value (SUVmax) on ^18^F‐fluorodeoxyglucose (FDG) PET/CT was an independent predictor of lymphovascular invasion [34]. Similarly, Foster et al. reported that FDG uptake parameters of IMAT, normalized for body habitus, were significantly associated with survival outcomes and serum biomarkers in patients with sarcoma [35]. Furthermore, low‐dose attenuation‐correction CT from PET/CT has been shown to provide sufficient image quality for body composition analysis, including IMAT segmentation [50]. However, despite its high clinical and research value, PET/CT and PET/MRI remain more expensive and less accessible than other conventional imaging modalities, limiting their routine use in clinical practice. A technical comparison of the principal imaging modalities for IMAT assessment and quantification, including their measurement approaches, spatial resolution, advantages and limitations, is provided in Table 2.

**TABLE 2:** Technical comparison of imaging modalities for the assessment and quantification of intermuscular adipose tissue.

Modality	Technique	Signal/contrast basis	Main measurement	Calculation	Spatial resolution^a^	Added value	Main limitation	Key references
CT	Attenuation‐based segmentation	X‐ray attenuation in HU	IMAT area/volume; muscle attenuation	Fat‐range HU thresholding within a defined ROI, followed by pixel/voxel counting	Protocol‐dependent; influenced by reconstruction and slice thickness	Enables opportunistic assessment using routine clinical CT	Ionizing radiation; attenuation varies with acquisition protocol and contrast phase	[25, 27, 50]
MRI	Conventional T1‐/T2‐weighted imaging	Signal‐intensity differences between fat and muscle	Visible IMAT area/volume	Visual or threshold‐based segmentation followed by voxel counting	Protocol‐dependent; influenced by matrix, field of view, and slice thickness	High soft‐tissue contrast; radiation‐free whole‐body assessment	Signal thresholds and segmentation methods are not fully standardized	[25, 28, 45]
MRI	CSE‐MRI/Dixon	Chemical‐shift difference between water and fat	Fat fraction/PDFF; segmented IMAT volume	Corrected fat signal relative to total fat and water signals; additional segmentation for IMAT volume	Protocol‐dependent; influenced by acquisition and reconstruction settings	Quantitative, repeatable water–fat assessment	PDFF measures tissue fat fraction rather than anatomical IMAT volume	[21, 25, 28, 31]
Ultrasound	B‐mode echo‐intensity analysis	Tissue backscatter and echogenicity	Echo intensity, muscle thickness, CSA, or IMAT index	Grayscale histogram or mean pixel‐intensity analysis	Dependent on probe frequency, depth, and beam orientation	Radiation‐free bedside assessment suitable for serial monitoring	Indirect and device‐dependent; affected by depth, settings, and tissue conditions	[25, 32, 47, 48, 49]
PET/CT or PET/MRI	Radiotracer‐based metabolic imaging	Radiotracer uptake combined with CT or MRI anatomy	SUVmean, SUVmax, or other uptake parameters	Tracer uptake measured within CT‐ or MRI‐defined ROIs	Protocol‐ and system‐dependent	Provides complementary information on glucose uptake and metabolic activity	Low adipose‐tissue FDG uptake may limit quantification; anatomical localization requires co‐registered CT or MRI	[33, 34, 35, 50]

Abbreviations: CSA = cross‐sectional area; CSE‐MRI = chemical shift‐encoded magnetic resonance imaging; CT = computed tomography; FDG = fluorodeoxyglucose; HU = Hounsfield unit; IMAT = intermuscular adipose tissue; MRI = magnetic resonance imaging; PDFF = proton density fat fraction; PET/CT = positron emission tomography/computed tomography; PET/MRI = positron emission tomography/magnetic resonance imaging; ROI = region of interest; SUVmax = maximum standardized uptake value; SUVmean = mean standardized uptake value.

^a^ Spatial resolution represents a protocol‐dependent typical clinical range rather than a fixed value.

### 3.2 Advanced Quantification Techniques

Early attempts to quantify IMAT relied mainly on manual or semi‐automated tracing of muscle boundaries on axial CT or MRI images. These methods were highly operator‐dependent and sensitive to acquisition parameters, often resulting in substantial inter‐observer variability. Subsequent studies revealed that IMAT quantification was also affected by imaging phase and patient demographics. Likewise, population‐based reference data demonstrated that IMAT levels varied significantly by sex and age, highlighting the limited comparability of traditional approaches across cohorts [51]. These findings emphasized the necessity for standardized, automated and reproducible IMAT quantification methods.

The introduction of deep learning has revolutionized IMAT assessment, moving it from subjective estimation to quantitative precision. The BioCompNet framework, which integrates dual‐parametric MRI and a hierarchical U‐Net, enables rapid and fully automated analysis of body‐composition compartments including IMAT [43]. In CT imaging, deep learning–based multilabel segmentation achieves high consistency with expert evaluation and maintains reliability even at markedly reduced radiation doses [52]. In musculoskeletal imaging, U‐Net segmentation applied to the Osteoporotic Fractures in Men Study (MrOS) cohort linked higher IMAT with poorer muscle strength and mobility [23]. Together, these developments have established IMAT quantification as a fast, reproducible, and clinically scalable technique. Before broad clinical adoption, automated IMAT segmentation models require external validation across institutions, imaging protocols, body regions, and disease populations, together with harmonized definitions of IMAT boundaries.

To ensure consistency across imaging centers and populations, researchers have increasingly adopted standardized ratios and density‐based parameters rather than absolute IMAT volumes. The use of metrics such as IMAT/total adipose tissue (TAT) and IMAT/SAT ratios has proven effective for survival prediction in metastatic prostate cancer [36], and higher IMAT fractions on CT angiography have been linked to greater amputation risk in peripheral arterial disease [37]. Integrating radiomic features from IMAT, VAT, SAT and skeletal muscle enables accurate prediction of hepatic decompensation and mortality in chronic liver and cardiovascular diseases [38]. Cardiometabolic studies further indicate that IMAT density correlates with major adverse cardiovascular outcomes and post‐transplant diabetes [39, 40]. Large‐scale MRI cohorts have also shown associations between intermuscular fat and chronic musculoskeletal pain [24]. Longitudinal investigations reveal that IMAT undergoes dynamic remodelling during disease progression and weight change, underscoring its potential as a long‐term biomarker for metabolic and musculoskeletal health [44]. In summary, to overcome measurement variability and enhance clinical generalizability, IMAT‐related research is shifting from isolated single‐slice measurements toward standardized volumetric, ratio‐based, density‐based and radiomics‐based metrics [22, 36, 37, 38, 39, 42, 43]. Volumetric analysis may better capture the anatomical heterogeneity of IMAT, whereas normalized indices such as IMAT/SAT, IMAT/TAT, or muscle fat fraction may improve comparability across individuals, scanners and body sizes [36, 37, 42]. These parameters have been demonstrated to hold significant prognostic and monitoring value across cardiovascular, oncological, metabolic and musculoskeletal diseases.

## 4 IMAT Distribution and Disease‐Specific Imaging Characteristics

Quantitative imaging studies have shown that IMAT demonstrates a distinct and heterogeneous distribution across the body, with predominant deposition in the lower extremities—especially the thigh and calf—followed by the paraspinal and shoulder‐girdle muscles, whereas the upper limbs contain minimal fat [45, 53, 54]. Whole‐body MRI and CT analyses consistently demonstrate that IMAT expands with advancing age [17], sedentary behaviour or neurological impairment [55], and metabolic abnormalities such as T2DM or impaired glucose tolerance [4, 56]. Paraspinal MRI validated against histologic reference confirms high measurement fidelity [45], supporting its utility for precise regional mapping of IMAT and other adipose depots.

Lower‐limb IMAT, particularly in the thigh, exhibits the strongest correlation with insulin resistance, independent of total or visceral adiposity [53, 54, 57]. Mechanistic studies indicate that IMAT secretes pro‐inflammatory and extracellular‐matrix‐remodelling factors that directly impair myocellular insulin signalling [57]. Conversely, SAT in the femoral‐gluteal region exerts metabolically protective effects, showing opposite relationships within the same anatomic plane [58]. In neuromuscular and musculoskeletal contexts, increased paraspinal IMAT correlates with reduced muscle attenuation and spinal instability [45, 55], while trunk or abdominal IMAT burden relates to diminished pulmonary capacity in the elderly [59] and adverse prognostic indicators in cancer populations [8, 60]. Muscular dystrophies represent a related but distinct neuromuscular context of muscle fat infiltration. In these disorders, fatty change is often dominated by intramuscular fibro‐fatty replacement rather than expansion of a clearly separable intermuscular fat depot; therefore, muscular‐dystrophy‐related fat infiltration should not be interpreted as directly interchangeable with IMAT. Nevertheless, evidence from skeletal muscle lipid and adipose‐tissue biology indicates that fatty infiltration can alter muscle structure, metabolism, and signalling pathways, while FAPs contribute to intramuscular adipogenesis during muscle degeneration and regeneration. These mechanisms support consideration of muscular dystrophies within the broader spectrum of disease‐specific muscle fat infiltration, while emphasizing that their subtype‐specific patterns should be evaluated with dedicated neuromuscular MRI studies [61]. These site‐specific findings collectively highlight that IMAT accumulation is anatomically selective and functionally consequential, reflecting local mechanical loading, vascularity, and metabolic microenvironments.

## 5 Disease‐Specific Imaging Features of IMAT

**FIGURE 2:**
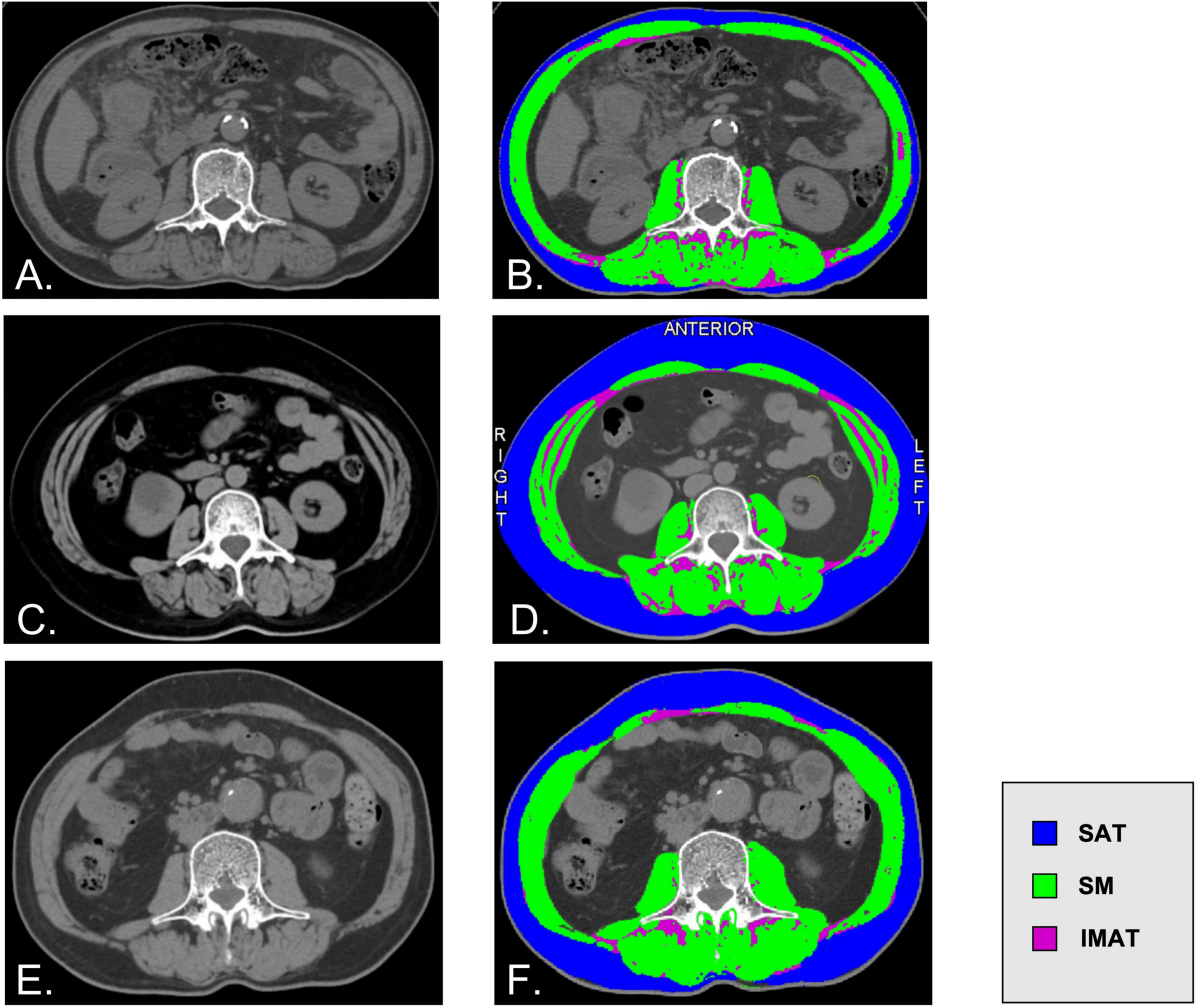
CT‐based IMAT imaging across metabolic and oncologic disease contexts. (A) Original axial CT image of a 52‐year‐old female with diabetes mellitus, obtained at a standard soft‐tissue window for body composition evaluation. (B) Corresponding segmented image with colour‐coded SAT, SM and IMAT. (C) Original axial CT image of a 72‐year‐old male with colorectal cancer, acquired at a soft‐tissue window for baseline body composition review. (D) Corresponding segmented image with colour‐coded SAT, SM, and IMAT. (E) Original axial CT image at the L3 level of a 63‐year‐old male with lung cancer, without apparent artefact or anatomic distortion. (F) Corresponding segmented image with colour‐coded SAT, SM and IMAT. Cross‐sectional areas were segmented and quantified using SliceOmatic software (Version 5.0; Tomovision, Montreal, Quebec, Canada). CT = computed tomography; IMAT = intermuscular adipose tissue; L3 = third lumbar vertebral level; SAT = subcutaneous adipose tissue; SM = skeletal muscle.

**FIGURE 3:**
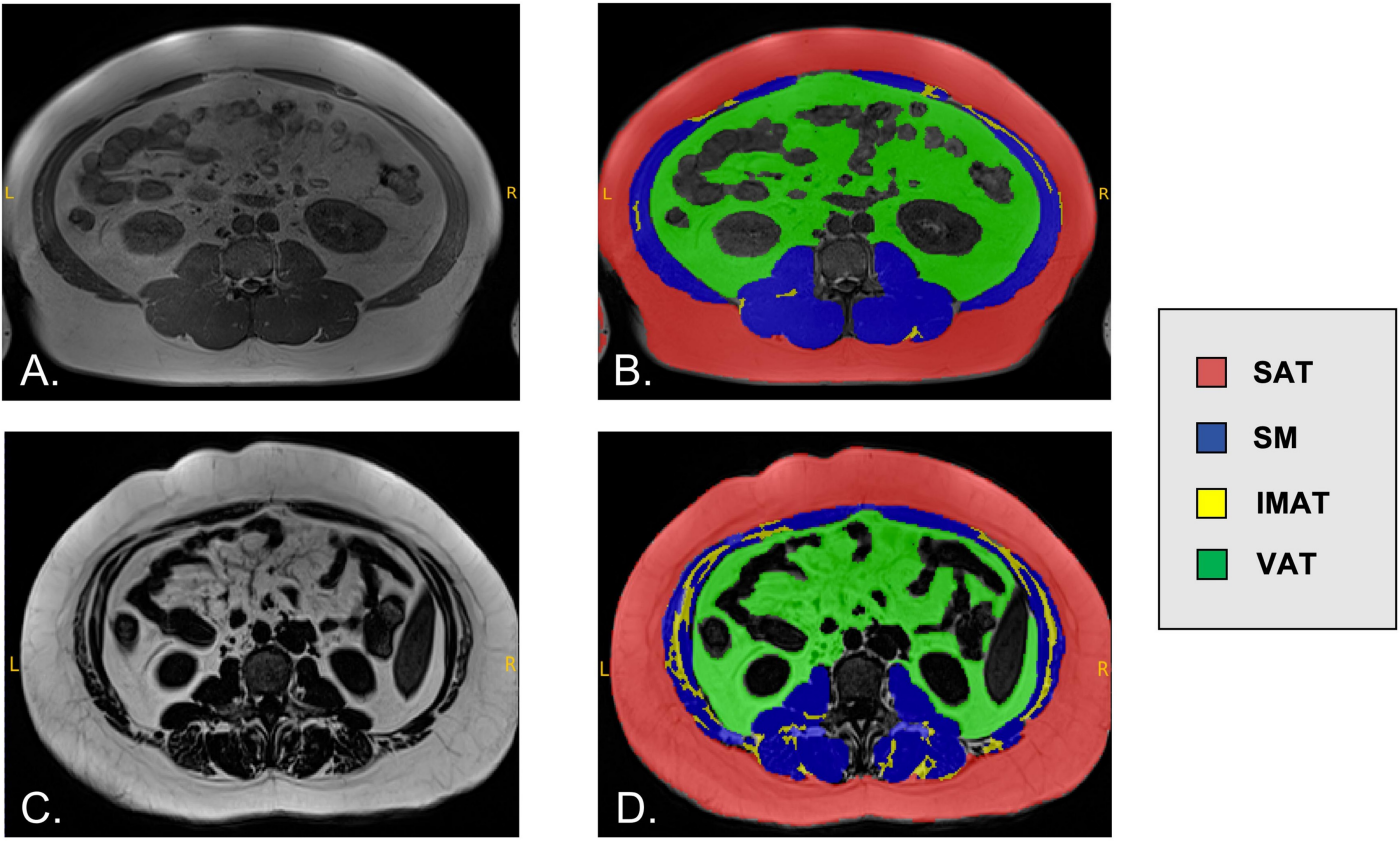
MRI‐based IMAT imaging in patients with comorbid type 2 diabetes mellitus and non‐alcoholic fatty liver disease. (A) Original axial MRI image of a 35‐year‐old male with type 2 diabetes mellitus and non‐alcoholic fatty liver disease, obtained at the abdominal level for body composition evaluation. (B) Corresponding segmented image with colour‐coded SAT (red), SM (blue), IMAT (yellow) and VAT (green). (C) Original axial MRI image of a 40‐year‐old female with type 2 diabetes mellitus and non‐alcoholic fatty liver disease, acquired at a comparable anatomical level for body composition assessment. (D) Corresponding segmented image with colour‐coded SAT, SM, IMAT and VAT. Both cases demonstrate prominent IMAT deposition interleaved within skeletal muscle bundles alongside expanded visceral adipose tissue, reflecting the adverse ectopic fat phenotype characteristic of concurrent metabolic dysfunction. Cross‐sectional areas were segmented using dedicated body composition software. IMAT = intermuscular adipose tissue; MRI = magnetic resonance imaging; SAT = subcutaneous adipose tissue; SM = skeletal muscle; VAT = visceral adipose tissue.

IMAT accumulation is consistently elevated in people with T2DM and obesity, and imaging evidence supports a dose‐dependent link with metabolic impairment (Figure 2A,B). Large CT cohorts demonstrate that higher trunk IMAT strongly associates with metabolic syndrome even after adjustment for age, lifestyle, and muscle mass [62]. Deep learning–based whole‐body MRI further shows that individuals with impaired glucose metabolism have higher IMAT and particularly higher skeletal muscle fat fraction which discriminates metabolic risk more effectively than IMAT alone [42] (Figure 3A–D). Recent large‐scale MRI studies further strengthen the metabolic relevance of IMAT in general populations rather than only in established diabetes cohorts. In more than 66 000 participants from the UK Biobank and German National Cohort, whole‐body MRI‐derived body‐composition z scores showed that abnormal skeletal muscle and adipose‐tissue profiles predicted incident diabetes and mortality beyond traditional cardiometabolic risk factors [63]. Although high VAT showed the strongest association with incident diabetes, inclusion of IMAT and skeletal muscle fat fraction helped characterize an adverse fat‐muscle phenotype that could not be captured by body mass index (BMI) alone [63]. These findings support the use of IMAT‐related imaging markers as part of metabolic risk stratification, especially for identifying individuals with apparently preserved body size but unfavourable ectopic fat distribution. Imaging studies help bridge mechanistic concepts with organ‐level phenotypes. Those with T2DM show greater ectopic hepatic and muscular lipids and reduced insulin sensitivity [64]. Hyperinsulinaemic euglycaemic clamp and microdialysis results indicate that impaired suppression of intramuscular lipolysis may raise local free fatty acid exposure and disturb glucose uptake. Reviews highlight that metabolic dysfunction reflects lipid intermediates such as diacylglycerols and ceramides and their intramuscular localization and turnover rather than absolute IMAT volume. Short‐term interventions reveal inertia of IMAT compared with VAT and muscle quality. Vigorous exercise improves insulin sensitivity and increases normal density muscle without clear IMAT reduction. Lipolysis inhibition during exercise enhances postprandial glycaemic control by shifting substrate use toward intramuscular triglycerides [65]. Sodium–glucose cotransporter 2 (SGLT2) inhibition reduces hepatic fat early in T2DM while changes in muscle fat lag behind [66]. Imaging therefore provides a sensitive readout of metabolic tissue remodelling and clarifies when IMAT acts as a risk signature of unhealthy muscle versus when lipid storage reflects adaptive oxidative capacity. To bridge these mechanisms with clinical manifestations, Table 3 summarizes disease‐specific IMAT imaging features, phenotypes, and prognostic implications across major organ systems.

**TABLE 3:** Disease‐specific imaging features and clinical implications of intermuscular adipose tissue.

System/disease type	Imaging modality	Main IMAT imaging features	Clinical/functional/prognostic associations	Key references
Metabolic diseases				
T2DM	CT; MRI	↑ Trunk IMAT; ↑ skeletal muscle fat fraction; ↓ muscle density	Strong predictor of insulin resistance and metabolic syndrome independent of BMI	[42, 62]
Metabolic risk in general populations	MRI	↑ IMAT z score; ↑ skeletal muscle fat fraction; adverse fat‐muscle phenotype	Cardiometabolic outcome prediction; risk beyond BMI and traditional factors	[63]
Postmenopause/insulin resistance	MRI; MRS	↑ Intramuscular lipids	↓ Whole‐body insulin sensitivity	[64]
Type 2 diabetes/NAFLD	MRS; clamp	Intramyocellular and extramyocellular lipids measured	Empagliflozin preferentially reduced liver fat; no differential improvement in muscle insulin sensitivity	[65, 66]
Neuromuscular diseases				
Neuromuscular disorders and sarcopenia	MRI	Muscle fat‐fraction imaging	Intramuscular fibro‐fatty replacement; distinct from IMAT; ↓ Muscle quality; ↑ muscle degeneration	[61]
Cardiovascular diseases				
Cardiometabolic risk factors	MRI	↑ Paraspinal IMAT; ↓ lean muscle mass	↑ Hypertension; ↑ atherogenic dyslipidemia; cardiometabolic risk clustering	[67]
CAD	CT; PET/CT	↑ IMAT; ↑ fatty muscle fraction; ↓ muscle density	↑ CAC; ↓ coronary flow reserve; predicts MACE	[40, 68, 69]
HFrEF	CT	↓ Muscle density indicating ↑ IMAT	Low muscle density/intramuscular fat was associated with incident HF/HFrEF	[70]
HFpEF	CT	↑ Thigh IMAT	↓ Exercise capacity; ↑ IL‐6, CRP, leptin; ↓ adiponectin	[71, 72]
Gastrointestinal and hepatobiliary cancers				
CRC	CT	↑ IMAT; ↑ IMAT/SAT ratio; ↓ radiodensity	↓ OS/DFS independent of stage; dynamic with cachexia; low radiodensity amplifies mortality risk	[60, 73, 74, 75]
HCC	CT	↑ IMAT; myosteatosis; muscle atrophy	Predicts early recurrence; MVI; ↓ survival; radiomics improves stratification	[76, 77, 78, 79, 80, 81, 82, 83, 84, 85, 86]
Pancreatic cancer	CT	↓ Muscle density = ↑ IMAT	↓ OS; visceral fat increases postoperative infection	[87]
Oesophageal cancer	CT; PET/CT	↑ IMAT; ↑ adipose SUVmax	↓ Survival; ↑ complications; sarcopenic obesity ↑ toxicity; prehabilitation mitigates loss; postoperative fat/muscle decline ↓ survival	[88, 89, 90, 91, 92, 93, 94, 95, 96, 97, 98, 99]
Gastric cancer	CT	↓ Radiodensity; ↑ low‐attenuation muscle; intermuscular fat metrics	Prognostic stratification; reliable IMAT metric	[100, 101, 102]
Thoracic/breast/prostate cancers				
Breast cancer	CT	Fat infiltration between muscle bundles; ↓ muscle density	↑ Chemotherapy toxicity; ↑ 1‐year mortality	[103, 104, 105, 106]
Prostate cancer	CT; PSMA‐PET/CT	Hypoattenuating strands in pelvic/thigh muscles	Poorer response to PSMA therapy	[36]
Lung cancer (NSCLC)	CT	Reticular low‐attenuation IMAT streaks (−190 to −30 HU)	Predicts ↓ survival and ↑ recurrence	[107, 108, 109]
Respiratory diseases				
COPD	CT	↑ Intermuscular fat; ↓ muscle density; diffuse streaks	GOLD progression; ↑ exacerbation risk; ↑ mortality	[110, 111, 112]
COPD‐associated quadriceps weakness	MRI	↑ Intermuscular fat; impaired muscle bioenergetics	Associated with reduced quadriceps strength	[113]

Abbreviations: BMI = body mass index; CAC = coronary artery calcium; CAD = coronary artery disease; COPD = chronic obstructive pulmonary disease; CRC = colorectal cancer; CRP = C‐reactive protein; CT = computed tomography; DFS = disease‐free survival; GOLD = Global Initiative for Chronic Obstructive Lung Disease; HCC = hepatocellular carcinoma; HFpEF = heart failure with preserved ejection fraction; HFrEF = heart failure with reduced ejection fraction; HU = Hounsfield unit; IL‐6 = interleukin‐6; IMAT = intermuscular adipose tissue; MACE = major adverse cardiovascular events; MRI = magnetic resonance imaging; MRS = magnetic resonance spectroscopy; MVI = microvascular invasion; NAFLD = non‐alcoholic fatty liver disease; NSCLC = non–small cell lung cancer; OS = overall survival; PET/CT = positron emission tomography/computed tomography; PSMA = prostate‐specific membrane antigen; SAT = subcutaneous adipose tissue; SUVmax = maximum standardized uptake value; T2DM = type 2 diabetes mellitus; ↑ = increased; ↓ = decreased.

In cardiovascular disease, imaging evidence consistently links skeletal muscle fat infiltration with both atherosclerotic burden and adverse outcomes. Recent population MRI evidence expands this association from patients with overt cardiovascular disease to earlier cardiometabolic risk states. In the German National Cohort, MRI‐derived paraspinal IMAT percentage was associated with hypertension and atherogenic dyslipidemia in 11 348 participants without known pre‐existing disease, while the combination of higher IMAT and lower lean muscle mass identified participants with the highest prevalence of cardiometabolic risk factors [67]. Similarly, whole‐body MRI‐derived reference curves from more than 66 000 individuals showed that high IMAT z‐score was associated with incident major adverse cardiovascular events, with a hazard ratio up to 1.54 compared with the middle z‐score category after multivariable adjustment [63]. These findings indicate that IMAT may serve not only as a marker of established cardiovascular morbidity but also as an imaging phenotype of subclinical cardiovascular risk. Abdominal IMAT quantified by CT is independently associated with coronary artery calcification in community cohorts, even after adjustment for BMI and other ectopic depots such as visceral and pericardial fat [68]. Studies using PET/CT similarly demonstrate that greater IMAT and a higher fatty muscle fraction correlate with impaired coronary flow reserve, reflecting coronary microvascular dysfunction, and predict major adverse cardiovascular events beyond traditional risk factors [40, 69]. Longitudinal observations further strengthen these associations. Higher intramuscular fat in the thigh, represented by reduced muscle density on CT, predicts incident heart failure, particularly with reduced ejection fraction, whereas intermuscular fat shows a less consistent association in this setting [70]. In obese patients with heart failure with preserved ejection fraction, greater thigh IMAT is linked to worse exercise capacity, indicating a potential role of peripheral muscle quality in limiting cardiorespiratory performance [71]. Mechanistically, inflammatory profiling suggests that increases in IMAT area and decreases in density are accompanied by higher IL‐6, CRP and leptin and lower adiponectin, consistent with a pro‐inflammatory environment that may promote atherosclerosis and vascular dysfunction [72]. Importantly, IMAT may be modifiable in selected populations. Moderate resistance training has been shown to reduce IMAT and improve several cardiometabolic risk indicators, supporting the potential value of imaging‐based monitoring with routine CT, MRI or PET attenuation‐correction imaging [39, 40, 114].

IMAT accumulation has emerged as a relevant imaging phenotype associated with tumour biology in colorectal cancer (Figure 2C,D). Across multiple cohorts of colorectal cancer patients, elevated IMAT or increased ratios such as IMAT/SAT consistently predicted adverse oncologic outcomes including significantly shorter overall and disease‐free survival independent of clinicopathologic factors [60, 73, 74, 75]. IMAT burden often coexists with impaired skeletal muscle quality, where low muscle radiodensity amplifies mortality risk and reflects a more aggressive metabolic phenotype [74]. Moreover, IMAT distribution changes dynamically with cancer progression and cachexia, indicating its potential role as a surrogate marker of tumour‐related catabolic stress [8]. Although evidence linking IMAT to treatment toxicities remains heterogeneous and findings in older adults suggest limited prognostic sensitivity, single‐slice and volumetric CT quantification methods show robust consistency, supporting IMAT as a practical radiologic biomarker for risk stratification [115]. Extending this framework to pancreatic malignancies may further clarify how regional ectopic fat infiltration contributes to systemic inflammation, sarcopenia and therapeutic vulnerability, highlighting IMAT‐derived phenotypes as meaningful imaging indicators in tumour assessment.

In hepatocellular carcinoma (HCC), multiple imaging studies have demonstrated that IMAT and myosteatosis, which are quantified through CT‐based indices and radiodensity, are consistently associated with adverse outcomes. Elevated IMAT or IMAT‐related markers predict early recurrence after hepatectomy [76, 77] and microvascular invasion (MVI) even in small tumours [78, 79], supporting a link with aggressive tumour biology. Meta‐analytic evidence further confirms that high intramuscular fat content correlates with worse survival and greater postoperative complications in cancers including HCC [80]. Beyond crude quantification, radiomics signatures derived from muscle and adipose compartments significantly improve risk stratification for postoperative recurrence and survival [76, 81]. Moreover, IMAT accumulation often coexists with muscle atrophy, forming a high‐risk metabolic phenotype with markedly impaired prognosis. These adverse prognostic effects remain robust across treatment modalities, such as surgery, immunotherapy, and transplantation, highlighting that IMAT‐related muscle degeneration reflects systemic vulnerability and reduced physiological reserve in patients with HCC [82, 83, 84]. Collectively, IMAT‐based imaging phenotypes add clinically relevant prognostic information that complements tumour staging and liver function assessment [85, 86], providing a framework to further understand ectopic fat‐related aggressiveness in hepatobiliary cancers. Building on this concept, IMAT has also demonstrated clinical relevance beyond hepatobiliary cancers. In pancreatic cancer, lower muscle attenuation, which reflects higher IMAT levels, has been linked to poorer overall survival, while greater visceral adiposity predisposed patients to postoperative infectious complications [87].

Comparable IMAT‐related phenotypes appear clinically meaningful in oesophageal cancer. After neoadjuvant therapy, changes in fat mass and visceral to subcutaneous adipose distribution correlated with circumferential margin positivity, while increased visceral adiposity independently predicted lower survival and coexisted with reduced muscle strength [88, 89]. PET‐based adipose tissue metabolic activity also reflected systemic aggressiveness, as higher subcutaneous adipose SUVmax associated with distant metastasis and shorter survival [90, 91]. Single‐slice and multi‐slice CT showed strong agreement in IMAT and adipose quantification, supporting robust applicability even in large‐scale assessments [92]. Context matters, since myosteatosis without systemic inflammation related to better survival in one chemoradiation cohort [93], and prognostic strength differed by histology in metastatic settings, being stronger in adenocarcinoma than in squamous disease [94]. Across treatment timelines, IMAT‐related changes quantified on CT indicate therapy tolerance and recovery potential. Prehabilitation during neoadjuvant treatment mitigated skeletal muscle loss and promoted visceral fat reduction, which corresponded to lower postoperative complication risk [95]. Long‐term follow‐up demonstrated marked visceral fat depletion and muscle loss after esophagectomy, and exceeding critical thresholds of postoperative decline associated with worse survival [96]. Sarcopenic obesity, combining visceral adiposity with low muscle mass, repeatedly emerged as a high‐risk condition linked to dose‐limiting toxicity during neoadjuvant chemotherapy [97], postoperative pneumonia and prolonged hospitalization [98], and inferior overall outcomes when compared with isolated adiposity or sarcopenia. Low subcutaneous adiposity itself was also associated with elevated mortality risk even after adjustment for disease severity [99], emphasizing the interconnected contributions of IMAT infiltration, adipose distribution patterns, and impaired muscle function to disease vulnerability. In gastric cancer, CT readily visualizes muscle fat infiltration and abnormal adipose distribution as key imaging phenotypes [100]. Increased IMAT typically appears as reduced skeletal muscle radiodensity and expansion of low‐attenuation muscle areas (LAMAs), indicating diffuse fatty replacement within muscle fibres [101]. Such changes are detectable even at baseline staging scans and often coexist with disproportionate visceral fat accumulation, which together depict a characteristic pattern of ectopic adiposity. In patients with obesity, more pronounced subcutaneous and visceral fat deposition can be accompanied by postoperative imaging findings such as intermuscular venous filling defects or pulmonary complications. Methodologically, single‐slice L3 CT quantification provides reliable measurements of IMAT and VAT and shows prognostic equivalence to multi‐slice volumetric analysis, although measurement accuracy may be slightly reduced in individuals with higher adiposity due to segmentation challenges [92]. Recent multicenter evidence further supports AI‐based CT body‐composition analysis, including intermuscular fat metrics, for sex‐specific prognostic stratification in gastric cancer [102].

In breast cancer, imaging demonstrates chemotherapy‐related infiltration of fat between muscle bundles and reduced muscle density on both ultrasound and histology validated CT changes [103]. Routine staging CT can identify elevated IMAT at the pectoralis region, enabling detection of muscle fat infiltration even when muscle area appears preserved [104]. L3 CT provides standardized quantification of adipose depots, yet young patients with advanced disease show disproportionately higher IMAT despite similar muscle bulk [105]. Chest CT visualizes thoracic IMAT adjacent to primary lesions and the heart, revealing regional fat redistribution that abdominal slices do not capture [106]. Across studies, imaging consistently reveals a shift from normal fascicle architecture to increased low‐attenuation compartments within muscle, reflecting local fat expansion beyond what BMI suggests. In metastatic prostate cancer, CT extracted from PSMA‐PET/CT highlights IMAT as discrete hypoattenuating strands embedded between pelvic and thigh muscle fibres, and patients with higher IMAT ratios exhibit visibly more fragmented muscle texture and inferior therapeutic outcomes [36]. Thigh CT in cancer survivors also displays focal intermuscular fat pockets separating muscle bundles, paralleling cognitive and functional decline even when muscle bulk appears preserved [116]. MRI demonstrates that in androgen‐deprived individuals, regional thigh IMAT shows limited reduction despite decreased abdominal fat, and axial images continue to show persistent fatty streaks interrupting normal muscle signal [117].

Extending that observation, thoracic CT characteristically depicts IMAT as streak‐like or reticular low‐attenuation regions interspersed between paravertebral and thoracic muscle bundles, disrupting the normal striated muscle pattern [107, 108]. These fatty streaks, typically ranging from −190 to −30 HU, are most evident along the erector spinae and intercostal planes on axial images and may extend toward the scapular or posterior thoracic wall [107, 108]. In early‐stage lung cancer, enlargement of these low‐density bands with reduced muscle attenuation represents thoracic myosteatosis (Figure 2E,F), which on preoperative CT correlates with poor postoperative survival and recurrence [107, 108]. Automated and AI‐based volumetric reconstructions further delineate IMAT as diffuse translucent layers within muscle groups that smoothly connect with subfascial fat, allowing precise three‐dimensional quantification with sub‐1% deviation from manual reference [109]. Beyond CT‐based structural characterization, ^18^F‐FDG PET/CT enables metabolic assessment of IMAT, and IMAT mean standardized uptake value (SUVmean) has been shown to be associated with unfavourable clinical outcomes [34]. Similar low‐attenuation intramuscular streaks have also been observed in chronic obstructive pulmonary disease (COPD) and cancer survivors, linking IMAT expansion on CT to diminished respiratory and cardiac function [110]. In COPD, CT‐based quantification consistently demonstrates that IMAT accumulates within the thoracic and abdominal musculature, paralleling disease progression across Global Initiative for Chronic Obstructive Lung Disease (GOLD) stages [110, 111, 112, 118]. Cross‐sectional and longitudinal imaging studies reveal that higher IMAT and LAMA [110] accompany reduced muscle density and impaired contractile bioenergetics, particularly in the quadriceps and pectoralis muscles [112, 113]. MRI spectroscopy confirms anaerobic metabolism in IMAT‐enriched muscles, indicating that fat infiltration disrupts oxidative capacity and contributes to functional decline [113]. Moreover, deep learning–derived chest CT biomarkers, such as pectoralis IMAT and extramyocellular fat index, predict adverse outcomes including pneumonia hospitalization and heart failure among COPD patients [111]. Collectively, these imaging findings support that CT‐visible IMAT expansion weakens respiratory muscle performance and is associated with increased risks of exacerbation and mortality [110, 118].

## 6 Clinical Translation of IMAT Imaging

### 6.1 Clinical Prognostic Implications of IMAT Imaging

Imaging‐based quantification of IMAT provides reproducible evidence linking fat–muscle imbalance to long‐term outcomes across metabolic, oncologic, and respiratory diseases. Recent population MRI cohorts further extend the clinical relevance of IMAT beyond oncology, showing that IMAT‐related phenotypes are associated with incident diabetes, major adverse cardiovascular events, and early cardiometabolic risk clustering [63, 67]. In metabolic disorders, CT and MRI directly visualize intramuscular fat infiltration preceding metabolic decline. For instance, baseline IMAT measured from chest CT independently predicted incident T2DM over 7 years, even after adjusting for BMI and waist circumference [118]. Similarly, abdominal IMAT quantified by AI segmentation was associated with diabetes and chronic pancreatitis development after acute pancreatitis, confirming its role as a metabolic risk depot. Quantitative IMAT mapping also supports perioperative assessment: increased thigh IMAT corresponded to poor glucose control after bariatric surgery and decreased insulin sensitivity in endurance training studies [117]. Accumulating evidence across oncology and aging research supports IMAT as a clinically relevant imaging biomarker. Elevated IMAT levels or IMAT‐based ratios have been consistently associated with adverse outcomes across multiple malignancies, including HCC, rectal cancer, prostate cancer, and breast and lung cancers, as well as functional decline in elderly cancer survivors [24, 104, 116].

Serial IMAT assessment may be useful for monitoring physiological adaptation, but interventional evidence remains heterogeneous. Exercise or resistance training may reduce IMAT or improve muscle quality in selected metabolic populations, with accompanying improvements in insulin sensitivity or cardiometabolic risk factors; however, other cohorts show persistent IMAT despite metabolic improvement, weight change, or rehabilitation [114, 117]. In prostate cancer receiving androgen deprivation therapy, metabolic parameters improved while IMAT remained stable, revealing persistent ectopic fat deposition undetected by laboratory tests [117]. Similarly, COPD patients retained high IMAT levels despite respiratory rehabilitation, marking poor exercise tolerance and higher mortality [118]. In cancer therapy, increases in IMAT during adjuvant chemotherapy predicted worse recurrence and treatment intolerance, whereas stable or reduced IMAT indicated therapeutic responsiveness [103]. Advances in deep‐learning segmentation have enabled automated IMAT detection from chest CT, achieving Dice coefficients of 0.87–0.95 and allowing large‐scale monitoring with minimal human input [111, 119]. These techniques transform IMAT into a dynamic biomarker for evaluating real‐time physiological response and optimizing therapy.

Integrating IMAT into predictive models enhances precision in risk assessment by uncovering hidden metabolic and oncologic vulnerability. Quantitative CT evidence shows that individuals with high IMAT, but normal BMI have significantly higher risks of metabolic syndrome and cardiometabolic events [118]. Beyond its standalone association with outcomes, IMAT metrics provide additive prognostic information beyond conventional clinicopathologic factors. Elevated IMAT‐to‐SAT ratios have been linked to higher postoperative recurrence and mortality in rectal and breast cancers [73, 104]. In HCC, incorporation of IMAT indices into survival models improved prognostic discrimination beyond tumour stage and serum biomarkers [83]. Similarly, AI‐based body composition analysis using the CT component of PET/CT, including IMAT quantification, has been associated with poor response to targeted or radioligand therapy in prostate and lung cancers [34, 36].

Collectively, these findings establish IMAT as a quantitative link between metabolic dysfunction and oncologic progression, providing a clinically relevant framework for personalized risk stratification and supportive‐care planning. In practical terms, IMAT metrics are best interpreted together with BMI, skeletal muscle area or density, tumour stage, performance status, inflammatory markers, and treatment context, rather than as isolated diagnostic thresholds [73, 74, 78, 85, 104]. This combined approach may help identify patients who appear clinically stable by conventional measures but have hidden metabolic or functional vulnerability that could influence prehabilitation, nutritional support, chemotherapy planning, postoperative surveillance, or rehabilitation strategies [14, 75, 95, 103, 104, 107]. Therefore, in current clinical workflows, IMAT assessment may be most feasible as an opportunistic analysis of imaging examinations already obtained for routine care, such as abdominal or thoracic CT, PET/CT, CT angiography, or routine oncologic staging scans, rather than as a stand‐alone indication for additional CT imaging [10, 29, 36, 37, 50, 107, 108, 109]. Dedicated MRI or Dixon/PDFF‐based protocols may be considered when repeated longitudinal monitoring is needed, particularly in younger patients or in settings where radiation exposure is a concern [20, 22, 28, 31, 42, 43]. Before broad clinical implementation, however, standardized acquisition protocols, segmentation thresholds, reference ranges, and disease‐specific decision cutoffs remain necessary [19, 25, 51, 52, 63].

### 6.2 Potential Actionable IMAT Patterns for Clinical Decision‐Making

Although universally accepted IMAT thresholds are not yet available, several clinically actionable patterns can be reasonably proposed from the existing literature. First, markedly elevated IMAT or low muscle radiodensity relative to age‐, sex‐ and disease‐specific reference distributions may identify individuals with adverse muscle quality who warrant further nutritional, metabolic, and functional assessment [51, 63]. In metabolic and cardiometabolic settings, high trunk or paraspinal IMAT, increased skeletal muscle fat fraction, or coexistence of high IMAT with low lean muscle mass may support referral for lifestyle intervention, resistance training, and cardiometabolic risk management, particularly when these findings occur despite a normal or non‐obese BMI [42, 62, 67, 114]. Second, in patients scheduled for major surgery, especially gastrointestinal, hepatobiliary, thoracic, or oesophageal cancer surgery, high IMAT, low muscle attenuation, sarcopenic obesity, or combined fat‐muscle imbalance should be interpreted as markers of reduced physiological reserve and may justify early dietitian referral, functional assessment, and structured prehabilitation rather than waiting for overt weight loss or low BMI [73, 75, 80, 87, 95, 98, 107]. Third, longitudinal worsening of IMAT‐related metrics, such as increasing IMAT burden, rising IMAT/SAT or IMAT/TAT ratios, or declining muscle radiodensity during neoadjuvant therapy, chemotherapy, rehabilitation, or surveillance, may indicate impaired treatment tolerance or incomplete recovery and should prompt reassessment of nutritional intake, exercise capacity, inflammatory status, and supportive care needs [36, 88, 95, 96, 103, 104]. In oncology, however, IMAT findings should not be used as a stand‐alone indication for chemotherapy dose reduction or regimen change; rather, they may inform closer toxicity monitoring and multidisciplinary treatment planning when combined with low muscle mass, sarcopenic obesity, poor performance status, organ dysfunction, or previous treatment intolerance [97, 104]. Importantly, whether reducing IMAT itself improves clinical outcomes remains unproven, because most intervention studies rely on imaging or metabolic surrogate endpoints rather than hard clinical outcomes. Current evidence therefore supports IMAT primarily as a risk‐stratification and treatment‐monitoring biomarker rather than as a validated therapeutic target.

## 7 Future Directions of Imaging Based IMAT Research

Quantitative imaging of IMAT has evolved from single‐slice manual tracing to large‐scale automated analysis. Early abdominal and thoracic CT studies demonstrated that IMAT quantification was feasible but exhibited substantial variability between readers and across imaging protocols. The implementation of deep learning‐based segmentation has markedly enhanced reproducibility and enabled standardized, high‐throughput assessment of muscle and adipose compartments across cancers and metabolic disorders [103, 109]. In hepatocellular and colorectal cancers, normalized IMAT ratios have provided more stable prognostic performance than conventional density‐based parameters [73, 78]. Despite these improvements, most current analyses remain dependent on standard‐dose CT confined to the L3 or T12 level, limiting population‐level and longitudinal applications. Low‐dose chest CT combined with AI reconstruction has recently shown reliable IMAT quantification with reduced radiation exposure [107, 109], while MRI using ultrashort echo time sequences allows detailed visualization of microscopic fat infiltration without ionizing radiation. Together, these developments are driving IMAT imaging toward clinical standardization and practical accessibility.

Multimodal imaging has begun to elucidate the biological roles of IMAT in both metabolic and oncologic conditions. CT and MRI consistently demonstrate that increased IMAT reflects impaired glucose regulation and heightened inflammatory signalling in metabolic disorders [66, 120]. In several cancers, increased or heterogeneous IMAT correlates with tumour aggressiveness and altered energy metabolism [34, 107]. MRI‐based metabolic mapping also indicates that local fat accumulation coincides with reduced oxidative capacity and altered muscle microstructure [100]. Combining imaging and molecular data could yield radiogenomic models that infer IMAT biology noninvasively [117]. Clinically, excessive IMAT predicts recurrence and poorer survival across malignancies [73, 78], and associates with reduced pulmonary and cardiac function in non‐oncologic disease [100, 110]. These findings position IMAT as a biomarker of muscle quality and systemic resilience. Future translation should focus on simple and reproducible measures such as ultrasound echo intensity or standardized CT/MRI indices to enable screening and personalized interventions in routine care. Key future directions and remaining unmet needs in IMAT imaging research are summarized in Table 4.

**TABLE 4:** Future directions, unresolved problems, and unmet needs in imaging‐based IMAT research.

Domain	Unresolved problem/unmet need	Future direction	Representative references
Standardized quantification	IMAT measurements vary by modality, anatomical level, scanner or device settings, segmentation method, and ultrasound‐specific factors such as ROI placement and echo‐intensity thresholds.	Harmonize CT, MRI, and ultrasound acquisition protocols, anatomical landmarks, segmentation definitions, and reporting standards, with additional standardization of ultrasound ROI placement and echo‐intensity thresholds.	[19, 25, 27, 29, 46, 49, 51]
Automated analysis	Manual and semi‐automated segmentation remain operator‐dependent and difficult to scale.	Validate AI‐based IMAT segmentation across centers, populations, and anatomical regions.	[22, 23, 43, 109, 111, 119]
Biological interpretation	It remains unclear whether IMAT is a causal mediator, adaptive lipid storage, or secondary marker of disease.	Combine imaging with inflammatory, metabolic, molecular, and functional assessments.	[1, 13, 26, 57, 64, 113, 120]
Clinical translation	Clinically actionable thresholds and validated intervention targets are not yet established.	Define disease‐specific cutoffs and test whether IMAT‐guided risk stratification or intervention improves outcomes.	[1, 25, 51, 63, 73, 80, 104, 114]

Abbreviations: AI = artificial intelligence; CT = computed tomography; IMAT = intermuscular adipose tissue; MRI = magnetic resonance imaging; ROI = region of interest.

## 8 Conclusion and Perspectives

Early clinical observations that linked IMAT to metabolic and inflammatory processes have evolved into a well‐established field of imaging‐based investigation. Modern quantitative imaging using CT, MRI, PET and ultrasound has transformed IMAT from a conceptual entity into a measurable biological marker that integrates information on metabolism, inflammation, and muscle degeneration. By providing consistent and reproducible measures of fat–muscle imbalance, imaging now enables early identification of metabolic dysfunction, prediction of therapeutic outcomes, and stratification of disease risk across multiple clinical settings.

As summarized in this review, IMAT can be reliably assessed using standardized CT‐ and MRI‐based methods, its distribution and imaging signatures vary across metabolic disease, age‐associated decline, and cancer‐related states, and these imaging‐derived markers hold growing promise for clinical risk stratification and treatment monitoring. Continued efforts to refine imaging protocols, harmonize analytical workflows, and integrate radiomic and molecular signatures will deepen mechanistic understanding of IMAT biology. Advances in AI, multimodal imaging, and low‐dose technologies are expected to expand accessibility and clinical applicability. Ultimately, integrating biological insight with precision imaging will position IMAT not only as a marker of disease burden but also as a dynamic indicator capable of guiding prevention, treatment and recovery across metabolic, oncologic and aging‐related conditions.

## Funding

This research received no specific funding.

## Conflicts of Interest

The authors declare no conflicts of interest.

## Data Availability

Data sharing not applicable to this article as no datasets were generated or analysed during the current study.
